# Effect of Savings on a Gas-Like Model Economy with Credit and Debt

**DOI:** 10.3390/e23020196

**Published:** 2021-02-05

**Authors:** Guillermo Chacón-Acosta, Vanessa Ángeles-Sánchez

**Affiliations:** 1 Departamento de Matemáticas Aplicadas y Sistemas, Universidad Autónoma Metropolitana Cuajimalpa, Vasco de Quiroga 4871, Ciudad de México 05348, Mexico; 2 Escuela Superior de Economía, Instituto Politécnico Nacional, Plan de Agua Prieta 66, Ciudad de México 11350, Mexico; vnne.as@gmail.com

**Keywords:** econophysics, savings propensity, geometric models

## Abstract

In kinetic exchange models, agents make transactions based on well-established microscopic rules that give rise to macroscopic variables in analogy to statistical physics. These models have been applied to study processes such as income and wealth distribution, economic inequality sources, economic growth, etc., recovering well-known concepts in the economic literature. In this work, we apply ensemble formalism to a geometric agents model to study the effect of saving propensity in a system with money, credit, and debt. We calculate the partition function to obtain the total money of the system, with which we give an interpretation of the economic temperature in terms of the different payment methods available to the agents. We observe an interplay between the fraction of money that agents can save and their maximum debt. The system’s entropy increases as a function of the saved proportion, and increases even more when there is debt.

## 1. Introduction

Econophysics brings together a set of theoretical and empirical achievements that come from applying well-known tools and physics, particularly from thermodynamics and statistical mechanics, to economics and financial markets. Its scope and applicability are still being discussed [1]. On the one hand, there are strong criticisms about the veracity of ideal hypotheses inherited from physical systems. However, recent evidence shows that well-known concepts such as Solow’s economic growth, and economic inequality in the sense of Piketty’s work, may arise from kinetic exchange models [2]. The truth is that this area has influenced financial economics studies for the last twenty years and can be considered a well-established current research branch [3].

The kinetic exchange gas-like models are inspired by molecular models of gases formed by colliding particles [4]. As the particles of a gas exchange energy during collisions, agents exchange a fraction of their capital under the hypothesis of the conservation of total money [5]. With this approach, it has been possible to reproduce some patterns that are observed in capitalist economic systems, such as the Pareto rule of wealth distribution [6]. These models’ main advantage, and the reason they have become attractive in various disciplines, is that their mathematical formulation and numerical implementation are very simple and straightforward [7]. They are so versatile that a wide range of practical economic and social interest situations have been addressed with them [8].

Through exchange models, some economic inequality indices have been explained and calculated from microscopic principles [9,10,11]. When studying the distribution of income and wealth with these models, different trends have been verified, such as the formation of classes [12,13]. Indeed, it has been seen that in the presence of labor unions, government policies to reduce inequality, or other solidarity factors of social protection, the corresponding wealth distribution changes to a bimodal distribution [14]. Although these distributions are rare and seem unrealistic, they have also been found when considering risky transactions and other stressful situations [15], such as an epidemic outbreak [16]. In order to simulate these theoretical models, it has been necessary to use different numerical and computational tools such as Monte Carlo methods for open systems [17] or classification methods for market behavior [18]. It has been shown how these exchange models could serve as optimization algorithms [19], so it would be interesting to use other techniques to study them [20,21].

Among the situations that can be addressed with these models, two are of interest in the present work. The first is that agents can incur debt when requesting credit from the bank. Credit and debt are introduced through a new variable that is different from the money coming from income, in such a way that it could minimize negative values, indicating the acquired debt [5]. The economic model, including credit and debt, was first studied by Viaggiu et al., using the tools of the statistical ensembles [22]. There, they adopt the Boltzmann–Gibbs distribution where energy is replaced by total money, including income, credit, and debt. This method was extended to studying markets and exchange economies through complex networks [23] and stock price formation processes from the order book [24]. This last system has also been addressed with the Boltzmann equation in a non-equilibrium situation [25]. The resulting aggregated economic variables could be related to macroeconomics in the same way that the gas particles’ microscopic energy gives rise to thermodynamics. It is worth noting that this idea of establishing a link between classical thermodynamics and economics is not new and was first suggested by Samuelson [26].

Just as in thermodynamics, where different microscopic interactions lead to measurable macroscopic effects, in agent models, it is possible to consider different transactions between agents and see their effect in analogous thermodynamic quantities. One of the interactions that can be modeled in this way, and we are interested in, is agent savings. In this model, saving propensity is defined as the fraction of an agent’s money λ that will not be spent during the transaction given by the corresponding exchange rule [27]. It is well known that saving agents’ income distribution follows the so-called Gamma distribution when the system reaches equilibrium. If the agents do not save, the Boltzmann–Gibbs distribution, which usually models the lower-middle class, is recovered. Indeed, Pareto’s law can be recovered for random savings within these models [28]. Furthermore, by considering that some agents have fixed savings and another sector has a random saving propensity, this leads to a distribution that has an exponential zone and another zone modeled by a power law, as observed in real economies [29]. The system of agents with saving propensity has also been studied through other approaches. In [30], López-Ruíz et al. introduce an analytical geometric model where agents’ money obeys an additive constraint that defines an *N*-dimensional equiprobability surface. The corresponding analog of the Hamiltonian contains the saving propensity as an exponent of a geometrical variable that can be identified with the monetary variable.

In this thermodynamic-like approach, the aggregate variables can tell us about the economic system’s behavior. For instance, the temperature of an economic system can be used as an index that indicates, on average, how the total money available is distributed among agents. For systems where agents can only pay with their given income, *T* turns out to be simply the average money per agent [5]. However, when debt is introduced or alternative means of payment are considered, this changes correspondingly [22]. On the other hand, by considering saving propensity, the economic temperature reduces by a factor that is a function of λ, analogous to the equipartition theorem of energy [30].

In this work, we study the thermostatistical properties of a kinetic exchange model that describes a simple closed economy with income, savings, credit, and debt. We analytically calculate the canonical partition function with the ensemble formalism, introducing the agents’ saving propensity through the geometric approach. From the partition function, we calculate the economic quantities that are analogous to the thermodynamic variables. In general, we observe an interaction between the fraction of money that agents can save and the debt that they can acquire. Specifically, we obtain the economic temperature and entropy. The economic temperature that we find can be read as the arithmetic means of two terms, the first is the average money per agent reduced by the saving propensity, and the second is related to the maximum overdraft. This second term reduces to *d* if no savings are present. Nevertheless, when λ≠0, it turns out to be non-trivially coupled with average money and the saving propensity. For a fixed temperature, debt induces an upper bound for the percentage that agents can save, such that, as the value of debt increases, agents save less. For entropy, we find that it has an increases in both temperature and savings. However, a minimum temperature appears for which the model is valid, which depends on the savings. Furthermore, these minimum temperatures and the entropy values change depending on the maximum debt.

This paper is structured as follows. In Section 2, we review the fundamental concepts of the geometric model for the distribution of the income for saving agents, and in Section 3, we review the ensemble theory for money, credit, and debt. In Section 4, we present the statistical ensemble for saving agents. We study the cases with and without debt and the corresponding limit for when the agents do not save money in their transactions, recovering the case studied by Viaggiu et al. [22], and Patriarca et al. [27], respectively. Finally, in the last section, we present a summary and discussion of the obtained results and possible future work routes.

## 2. Microscopic and Geometric Models for a System of Saving Agents

Let us consider a simple, discrete, closed economy model in which *N* agents can exchange money in pairs. In the beginning, each agent has the same amount of money, say M/N, where *M* is the total money in the system, so the initial distribution of capital is uniform. Then, at each time step, a pair of agents (i,j) randomly chosen begin to interact with each other, where i≠j, and i,j=1,2,…,N.

**Figure 1:**
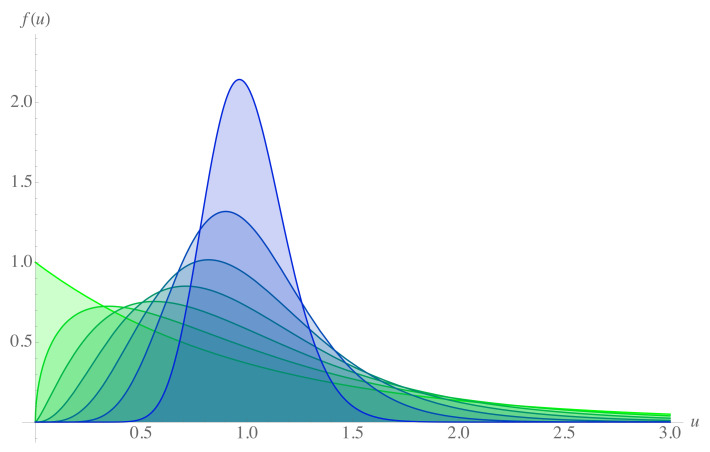
Gamma distribution function for distribution of money Equation (3) for different values of the saving propensity λ. The green curve corresponds to λ=0, the saving factor increases as it tends to the blue, so the darker blue curve corresponds to λ=0.9.

During the exchange, the capital of each agent changes following the next exchange rule constrained to the fact that the total money is conserved.

(1) $$\begin{array}{c} u_{i}^{\prime }=u_{i}+\Delta u,\;u_{j}^{\prime }=u_{j}-\Delta u, \end{array}$$

where $u_{i}$ and $u_{i}^{\prime }$ is the money of agent *i* before and after a transaction, respectively. The amount exchanged Δu is taken randomly and depends on the details of the transaction, for instance, for constant saving propensity λ is

(2) $$\Delta u=\left(1-\lambda \right)\left[\epsilon u_{j}-\left(1-\epsilon \right)u_{i}\right],$$

where ϵ is a uniformly distributed random variable. In this case, during the transaction between agents *i* and *j*, the money that can be reallocated is reduced by 1−λ, corresponding to the fraction of the initial capital each agent has decided to use in the exchange. For this model, the agents’ equilibrium distribution has been studied both analytically and numerically, and it depends on the value of the saving propensity parameter. Specifically, simulations point to a Gamma distribution for the money [27]

(3) $$\begin{array}{c} f\left(z\right)dz=\frac{z^{n-1}}{\Gamma (n)}e^{-z}dz, \end{array}$$

where $z=\frac{nu}{\langle u\rangle }$, 〈u〉 is the average money and

(4) $$n\left(\lambda \right)=\frac{1+2\lambda }{1-\lambda },$$

is the shape factor of the Gamma distribution, which, in this context, is a function of the saving propensity. For λ=0, where there is no saving criterion, Equation (3) reduces to the Boltzmann–Gibbs distribution [5]. Distribution (3) for increasing values of the saving propensity can be seen in Figure 1.

**Figure 2:**
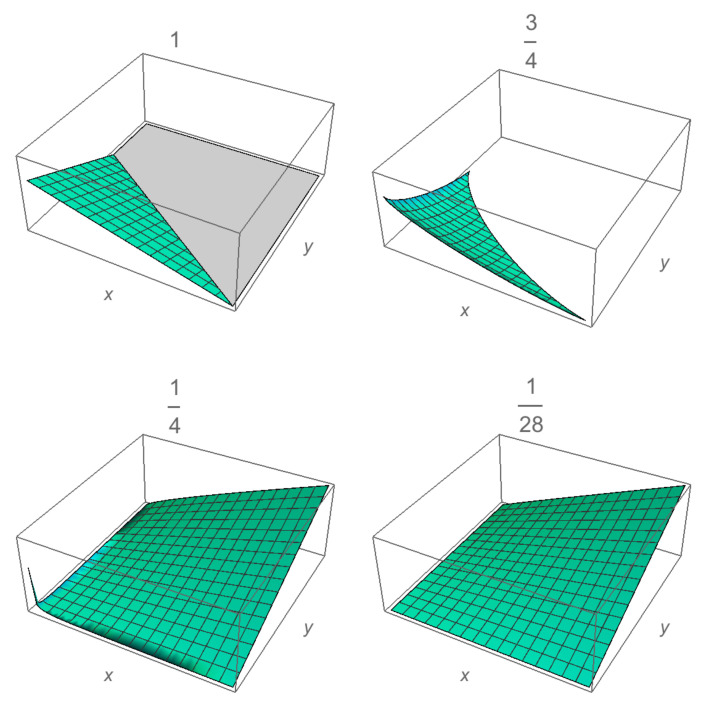
Constriction surfaces Equation (5) for three agents with M=1, for different values of b=1,3/4,1/4,1/28, which correspond to a saving propensity of λ=0,0.1,0.5,0.9, respectively.

The same distribution can be obtained from a geometric perspective [30]. Let be a set of positive variables ${\left\{x_{i}\right\}}_{i=1,\ldots ,N}$ satisfy the constraint

(5) $$\begin{array}{c} x_{1}^{b}+x_{2}^{b}+\ldots +x_{N}^{b}=\sum_{i=1}^{N}x_{i}^{b}\leq M, \end{array}$$

with *b* a positive real constant and *M* the total money. The equality in (5) defines a symmetrical surface, it also defines a transaction between agents. Here, *x* is an internal geometrical variable related to the capital per agent through the probability density [30]. The probability f(x)dx of finding an agent with generic coordinate *x* is proportional to the volume $V_{N-1}\left({\left(M-x^{b}\right)}^{1/b}\right)$ of all the points contained into the (N−1)—dimensional symmetrical region limited by the constraint $\left(M-x^{b}\right)$. A visualization of these surfaces for the case of three agents is shown in Figure 2. Thus, by considering the normalization condition, the distribution function is $f\left(x\right)=\frac{V_{N-1}\left({\left(M-x^{b}\right)}^{1/b}\right)}{V_{N}\left(M^{1/b}\right)}$. By assuming that the volume is proportional to the radius of the region, and for large *N*, it is possible to find the same Gamma distribution (3) for the desired probability, with $z=\frac{x^{b}}{b\;\langle x^{b}\rangle }$. Thus, by comparing both expressions, we find

(6) $$\frac{u}{\langle u\rangle }=\frac{x^{b}}{\langle x^{b}\rangle }.$$

where $b=b\left(\lambda \right)=n{\left(\lambda \right)}^{-1}$. Note that in the particular case when b=1, corresponding to no savings, *x* is exactly the money of the agents. In this way, saving propensity appears in the constriction through the power of the geometric variables. This model will allow for a Hamiltonian formulation proposal that we will use when defining the saving agents’ ensemble.

## 3. Statistical Ensembles for Agents with Credit and Debt

As noted by Viaggiu et al. [22], to build an ensemble, it is only necessary to have a conservation law. In their case, they introduce the total money in the system as a conserved quantity, which depends on two possible variables—*u* the money of each agent coming from income and *v*, which is a monetary variable whose positive values correspond to the credit obtained by the agent, and its negative values to the acquired debt. Therefore, in general

(7) $$m\left(u,v\right)=\sum_{i=1}^{N}\left(u_{i}+v_{i}\right).$$

The canonical partition function is introduced as follows [22]

(8) $$Z=\int \int d^{N}u\;d^{N}v\;e^{-\frac{m(u,v)}{T}},$$

where *T* is the economic temperature related to the average money per agent but depends on agents’ interactions. With this definition of the partition function, it is possible to find the thermodynamic variables in the same way as in equilibrium statistical mechanics. In particular, entropy has the same interpretation, being proportional to the number of microscopic configurations of the system; therefore, the equilibrium state corresponds to the configuration that maximizes the entropy.

In order to interpret the meaning of economic temperature, let us briefly review three cases. Firstly, let us consider the simple case $M\left(u\right)=\sum_{i}u_{i}\;$, so the integral of the partition function (8) is simply

(9) $$Z=V_{v}^{N}T^{N},$$

where $V_{v}$ is the integral over the *v*-variable, which is constant and depends only on the domain of *v*, and can be interpreted as the maximum credit accessible in the system. From the usual thermodynamic relationship for the internal energy, namely

(10) $$M=T^{2}\frac{\partial }{\partial T}\ln Z=NT,$$

we can obtain the system’s total capital and the economic temperature as the mean capital per agent T=M/N.

As mentioned above, in these gas-type models, debt can be modeled as negative money; this appears when an agent does not have enough capital and borrows a certain amount from a bank, which in this model will not charge interest, so the agent’s balance, in the end, becomes negative [5,22]. Let us now consider the function money for credit and debt as $m\left(u,v\right)=\sum_{i}\left(u_{i}+v_{i}\right)\;$, where $v_{i}\in \left[-d,\infty \right)$ with d⩾0. Thus, if $v_{i}$ is positive, the agent has available credit that he can use in the same way as $u_{i}$, while if $v_{i}$ is negative, he has borrowed money previously, with maximum debt *d*, that for simplicity, we consider the same for all agents. However, a more realistic situation would be for the bank to decide which limit to impose on each agent based on their income and credit score. The partition function and thermodynamic quantities can be calculated with the corresponding limits, and the result is as follows:

(11) $$Z=T^{2N}e^{\frac{Nd}{T}},\;T=\frac{1}{2}\left(\frac{M}{N}+d\right).$$

Let us explore a couple of features. In this case, the temperature is not simply the average money per agent, but it is distributed among the different payment alternatives, namely, the agent’s money and his approved credit.

Economic temperature *T* can be thought of as an index that relates the average money to an agent’s debt capacity. For instance, if M/N<d then T/d<1, which indicates that in such an economy, the agents could not cover a debt *d* on average. Indeed, this ratio is bounded when the average money is much lower than the amount of credit; then, T/d tends to 1/2. In the case that M/N∝d, then T/d≈1, which is the limit of the debt that could be covered. Then, for an economy to have no problem with a credit amount *d*, we should look for at least T/d>1, which implies that, on average, M/N>d. Indeed, when we ask for M/N≫2d, then T/d≫1, there will be enough liquidity to cover the debt in this regime.

On the other hand, recalling the standard interpretation of the partition function *Z* as the number of accessible microstates of the system for a given temperature, according to the expression (11), the number of accessible states grows exponentially with the ratio d/T. In fact, given those mentioned above, the more microscopic states are available for the system, the more difficulty the agents will have to pay the debt.

We can go further, removing the credit from the agents and leaving them with the debt *d*, which can be done if v∈[−d,0), d⩾0. In this case, the partition function and the thermodynamic quantities are as follows:

(12) $$Z=T^{2N}{\left(e^{d/T}-1\right)}^{N},\;M=2NT-\frac{Nd\;e^{d/T}}{e^{d/T}-1}.$$

Although apparently in the limit d/T≫1, the expressions of the previous case are recovered, this case is not valid in the corresponding regime, in addition to having the aforementioned problems. However, if the debt is small, an expansion of the exponential can be performed for d/T≪1, which implies that

(13) $$Z\approx T^{N}d^{N}\left(1+O\left(\frac{d}{T}\right)\right),\;M\approx N\left(T-\frac{d}{2}+O\left(\frac{d^{2}}{T^{2}}\right)\right).$$

In this limit, economic temperature goes as

(14) $$T≃\frac{M}{N}+\frac{d}{2}+\ldots ,$$

which is consistent with M/N≫d/2. The temperature of the system increases a little as long as the debt is much less than twice the average capital.

## 4. Statistical Ensambles for Money, Credit and Debt with Saving Propensity

In this section, we study the effect of the saving propensity on thermodynamic quantities, particularly in terms of economic temperature, entropy, and in the partition function. Given the Hamiltonian formulation in Section 2, we can construct different money functions to calculate the corresponding thermodynamics and partition functions.

### 4.1. Case 1: Money and Savings

Let us consider directly the money function (5) and calculate the partition function simply

(15) $$Z=\int \int d^{N}x\;d^{N}y\;e^{-\frac{1}{T}\sum_{i}x_{i}^{b}}=V_{y}^{N}{\left(\int e^{-\frac{\;x^{b}}{T}}dx\right)}^{N},$$

where *b* is given by the inverse of Equation (4). This integral can be easily related to the Gamma function through a variable change, such that

(16) $$Z=V_{y}^{N}T^{\frac{N}{b}}\Gamma {\left(\frac{1}{b}+1\right)}^{N}.$$

With the use of the definition (10), we can determine the economic temperature

(17) $$M=\frac{NT}{b}\;,\;\Rightarrow \;T=b\frac{M}{N}.$$

**Figure 3:**
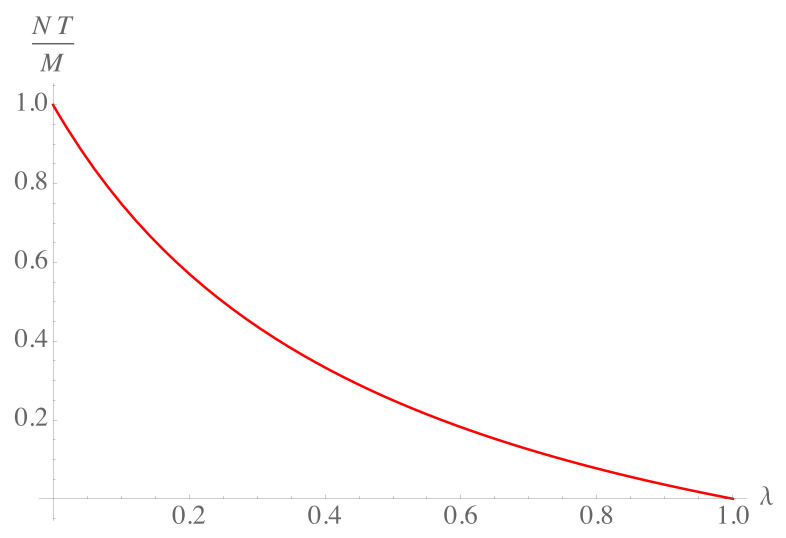
Plot of the ratio of economic temperature and the average money per agent as function of the saving propensity.

The expression (17) resembles the equipartition theorem of energy, which states that each microscopic degree of freedom contributes by a term proportional to *T* to the total energy. In this case, we can say that each agent contributes to the total money an amount proportional to the economic temperature by a coefficient that is a function of the saving propensity, Equation (4), such that 0≤b≤1. The ratio between temperature and the money per agent decreases as the fraction of money saved increases, see Figure 3.

For the ideal gas, the equivalent ratio of *T* by the energy per particle is a constant equal to 2/3, which depends on the functional form of the microscopic kinetic energy and the dimension. Expression (17) was previously obtained by Patriarca et al. [27], where $b^{-1}$ is interpreted as an effective dimension.

**Figure 4:**
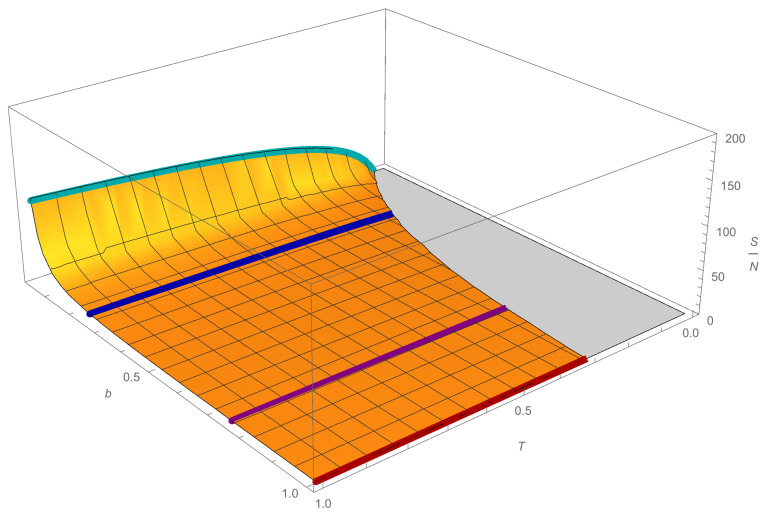
Plot of the ratio between the entropy and the number of agents (18) as a function of the temperature *T* and the parameter *b*, with $V_{y}=1$. The curves are presented for the values of b=1 (red), 3/4 (purple), 1/4 (blue), 1/28 (cyan), which correspond to a saving propensity of λ=0,0.1,0.5, and 0.9, respectively.

It is also possible to obtain the entropy of the system as typically done in thermodynamic systems TS=M+TlnZ, with which the following expression is obtained:

(18) $$S=\frac{N}{b}\left[\ln T+1+\ln\left(1+\frac{1}{b}\right)\right]+N\ln V_{y},$$

where there is no emphasis on the indistinguishability of the agents. The expression (18) is plotted in Figure 4, where, for each fixed value of λ, an increasing logarithmic behavior in *T* can be seen, as occurs in thermodynamics with the well-known Sackur–Tetrode formula. However, in this case, we see that the entropy begins to grow from a certain minimum temperature that changes according to the agents’ saving capacity; the more they save, the lower the minimum temperature is. Indeed, we can also observe that the more the agents save, the more rapidly the entropy increases.

### 4.2. Case 2: Savings, Money, Credit and Debt

To introduce credit and debt, we must add to the money constraint (5) an additional term with monetary units in a similar way as was done in Equation (7). Let us call $y^{b}$ the corresponding geometric variable, whose interpretation is similar to that of the Equation (6) and must satisfy $y\in [-d^{1/b},\infty )$. Here, we can identify *d* again as the maximum debt. Therefore, for the money function $m\left(x,y\right)=\sum_{i}\left(x_{i}^{b}+y_{i}^{b}\right)$, we can calculate the partition function as follows:

(19) $$Z=\int \int d^{N}x\;d^{N}y\;e^{-\frac{1}{T}\sum_{i}\left(x_{i}^{b}+y_{i}^{b}\right)}={\left(\int_{0}^{\infty }e^{-\frac{\;x^{b}}{T}}dx\right)}^{N}{\left(\int_{-d^{1/b}}^{\infty }e^{-\frac{\;y^{b}}{T}}dy\right)}^{N}.$$

The first integral is, again, a gamma function, while the second one can be rewritten as the upper incomplete gamma function $\Gamma (b^{-1},zdT^{-1})$, where $z=e^{i\pi b}$. This last term appears due to the negative lower integration limit introduced by the debt. However, this function can be analytically continued to complex numbers, maintaining many of its real-valued counterparts’ properties. In particular, it satisfies [31]:

$$\Gamma \left(a,rz\right)=z^{a}\Gamma \left(a,r\right)+\left(1-z^{a}\right)\Gamma \left(a\right).$$

With this property together with the relation γ(a,x)+Γ(a,x)=Γ(a), with γ(a,x) the lower incomplete gamma function [31], the partition function can be written as

(20) $$Z={\left\{\frac{T^{2/b}}{b^{2}}\Gamma \left(\frac{1}{b}\right)\left[\Gamma \left(\frac{1}{b}\right)+\gamma \left(\frac{1}{b}\;,\frac{d}{T}\right)\right]\right\}}^{N}.$$

By taking the derivative of the partition function, we find the total money of the system

(21) $$M=\frac{2NT}{b}\left[1-\frac{b}{2}{\left(\frac{d}{T}\right)}^{1/b}\frac{e^{-d/T}}{\Gamma \left(\frac{1}{b}\right)+\gamma \left(\frac{1}{b}\;,\frac{d}{T}\right)}\right].$$

This expression is much more involved than in the previous cases; particularly, we realize that we need to solve a transcendent equation to obtain the temperature. This implies that economic temperature will no longer be directly proportional to the mean capital per agent. Nevertheless, this also occurs in other physical systems, consider, for example, the relativistic gas [32], where the proportionality between temperature and energy can only occur in the non-relativistic and ultra-relativistic limits, for low and high temperatures, respectively. For intermediate values of *T*, from a certain critical temperature [33], it is not possible to have a simple relationship between these variables.

In order to have a more manageable expression of Equation (21), let us consider the same expansion as in Section 3 for d≪T. In such a case, the first terms of the expansion of the exponential can be considered, and for the incomplete gamma function, the first terms of the following series [31]

(22) $$\gamma \left(a,x\right)=x^{a}\sum_{k=0}^{\infty }{\left(-1\right)}^{k}\frac{x^{k}}{k!(k+a)},\;\mathrm{for}\;x\ll 1.$$

Hence, we obtain the following:

$$M≃\frac{2NT}{b}\left\{1-\frac{b}{2\Gamma \left(\frac{1}{b}\right)}{\left(\frac{d}{T}\right)}^{1/b}\left[1-\frac{d}{T}+\frac{1}{2}{\left(\frac{d}{T}\right)}^{2}-\frac{b}{\Gamma \left(\frac{1}{b}\right)}{\left(\frac{d}{T}\right)}^{1/b}\left(1-\frac{d}{T}\frac{b+2}{b+1}+\ldots \right)+\ldots \right]\right\},$$

(23) $$M≃\frac{2NT}{b}\left\{1-\frac{b}{2\Gamma \left(\frac{1}{b}\right)}\left[{\left(\frac{d}{T}\right)}^{\frac{1}{b}}-{\left(\frac{d}{T}\right)}^{\frac{b+1}{b}}+\frac{1}{2}{\left(\frac{d}{T}\right)}^{\frac{2b+1}{b}}-\frac{b}{\Gamma \left(\frac{1}{b}\right)}{\left(\frac{d}{T}\right)}^{\frac{2}{b}}+\ldots \right]\right\}.$$

First, we see that in the case where the agents do not save, i.e., b=1, Equation (23) reduces to M≈2NT−dN, which is precisely Equation (11) that was obtained by Viaggiu et al. [22]. For b≠1, we can consider just the first term of the previous series

(24) $$M≃\frac{2NT}{b}\left[1-\frac{b}{2\Gamma \left(\frac{1}{b}\right)}{\left(\frac{d}{T}\right)}^{\frac{1}{b}}\right].$$

**Figure 5:**
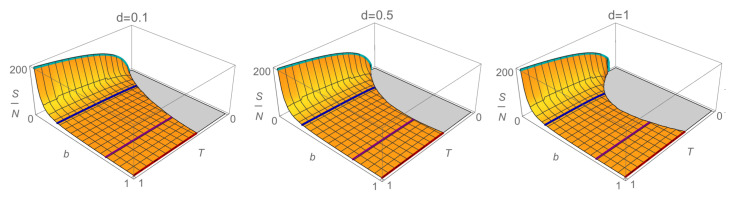
Plot of the ratio between the entropy and the number of agents (25) as a function of the temperature *T* and the parameter *b* for d=0.1,0.5,1. We note a change in the behavior of *S* as *d* grows. Again, the curves are for b=1 (red), 3/4 (purple), 1/4 (blue), 1/28 (cyan), and correspondingly λ=0,0.1,0.5, and 0.9.

With this expression, it is possible to obtain an approximate expression for the entropy in the same way as in the case of the previous subsection

(25) $$\frac{S}{N}≃\frac{2}{b}\left[1-\frac{b}{2\Gamma \left(\frac{1}{b}\right)}{\left(\frac{d}{T}\right)}^{\frac{1}{b}}\right]+\frac{2}{b}\ln T-2\ln b+2\ln\Gamma \left(\frac{1}{b}\right)+\frac{b}{\Gamma \left(\frac{1}{b}\right)}{\left(\frac{d}{T}\right)}^{1/b},$$

where the last term corresponds to the approximation of the incomplete gamma function with the leading term of (22). In the case b=1, Equation (25) reduces to S≈2NlnT+2N, obtained in [22]. We show in Figure 5, three graphs of *S* for different values of *d*. It can be seen that the behavior is qualitatively similar to that of Figure 4. However, we note that when d≠0, entropy grows faster as *b* decreases, or when saving increases. Furthermore, as *d* increases, the behavior of the minimum *T* changes in each case.

To interpret the economic temperature, in this case, consider Equation (24) and solve for *T* as follows:

(26) $$T≃\frac{Mb}{2N}\left[1+\frac{b}{2\Gamma \left(\frac{1}{b}\right)}{\left(\frac{d}{T}\right)}^{\frac{1}{b}}\right].$$

Let us realize that *T* still appears on the right-hand side of the above expression. However, as a first approximation, we can consider $T_{0}∼Mb/2N$, and replace it into Equation (26), to have an iterated solution, as shown below.

(27) $$T\approx \frac{Mb}{2N}+\frac{d^{1/b}}{2\Gamma \left(\frac{1}{b}\right)}\;{\left(\frac{Mb}{2N}\right)}^{1-\frac{1}{b}}=\frac{1}{2}\left[b\frac{M}{N}+\frac{b\;d^{1/b}}{\Gamma \left(1+\frac{1}{b}\right)}\;{\left(\frac{Mb}{2N}\right)}^{1-\frac{1}{b}}\right].$$

In this case, we can keep the interpretation of *T* as the arithmetic means of two payment methods, the first, given by the average money per agent reduced by a factor dependent on the savings propensity, while the second term corresponds to a non-trivial function of the debt and average money, which reduces to *d* in the case of no savings.

**Figure 6:**
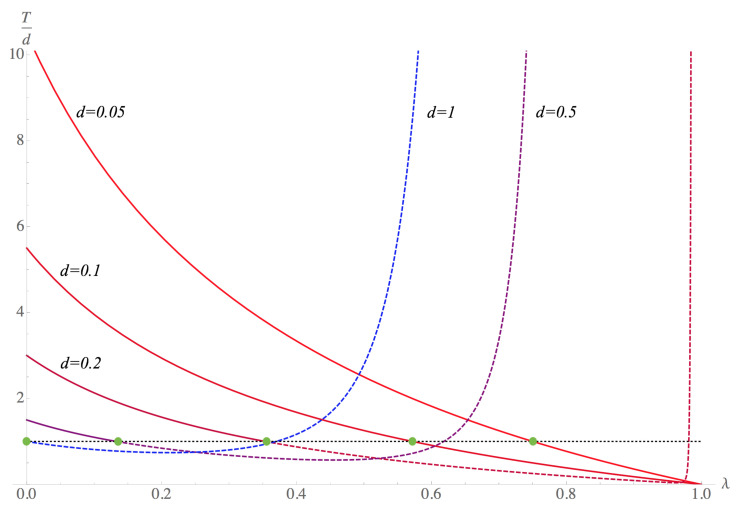
Graphic of the ratio $\frac{T}{d}$ from Equation (27) as function of savings propensity λ for unitary average money per agent. The values of d= 0.05, 0.1, 0.2, 0.5, 1. The approximation fails for T=d, so the dashed branches of the graph have no interpretation in the model.

To analyze the behavior of the temperature given by Equation (27), it is convenient to study the ratio T/d, since such an approximation is valid when this ratio is much greater than one. In Figure 6, the ratio T/d is plotted as a function of the saving propensity for the particular case in which the mean money per agent is equal to 1, in monetary units. For the case in which debt *d* is small compared to one, the savings decrease the relation T/d as λ increases. When T=d, the approximation is no longer valid, this defines a $\lambda_{max}$, which is the maximum percentage that an agent can save. As the value of debt increases, $\lambda_{max}$ decreases, i.e., agents save less. When d=1, the approximation is not valid for any λ. It is interesting to see that as *d* increases, the ratio T/d increases for high values of λ. This behavior has no meaning in terms of the model, although mathematically, it is consistent with the application regime. To summarize, as *d* increases, the agents’ savings capacity decreases.

## 5. Conclusions

In this work, we applied the statistical ensemble formalism to a geometric model of agents to study the effect of saving propensity on a system with money, credit, and debt. This formalism allowed us to obtain analogous thermodynamic variables. We studied the system’s economic temperature, which is an index that relates the average money with the agents’ debt capacity. The exact expression is given by the solution of Equation (21). In the case T≫d, which corresponds to a system where agents can pay their debt, it was possible to find an approximation for the economic temperature expressed as Equation (27); that is, the arithmetic mean between the different payment methods, namely, cash from income and the requested credit with a specific debt limit. The savings propensity modifies each term. On the one hand, the term associated with the average money per agent follows an analogous equipartition theorem and is reduced by a factor b(λ), while the term associated with the debt is coupled with the average money and the savings propensity in a non-trivial way, but reduces to *d* when there is no saving. We show the behavior of Equation (27) in Figure 6, where we see that the ratio between *T* and *d* decreases as savings propensity grows to a maximum value that depends on *d*. The entropy of the saver agent system when debt is present was also calculated. In general, we can say that entropy increases as savings increase, starting from a minimum temperature that depends on *b*, for which the entropy is greater than zero. In the case of having debt, the entropy increases even more, and the variations in the minimum temperature when changing *b* vary according to the different values of *d*. Although these analogous thermodynamic variables are not in general use in economics, economic temperature or entropy could certainly serve as indicators of how agents’ decisions affect the collectivity and, therefore, suggest how economic systems behave.

As mentioned in the introduction, there are some criticisms of these models; for example, ideal hypotheses limit their scope. However, the models have the advantage that their mathematical formulation is easily generalizable. Indeed, this formalism of statistical ensembles can be extended to more complicated or realistic cases or with different kinds of microscopic interactions. For example, in a most realistic case in which the bank establishes the credit limit according to each agent, the integrals in Equation (19) for calculating the partition function give a product of incomplete gamma functions with different arguments each, and the calculation becomes more involved. The difference between having an individual and a collective debt limit has recently been studied in the context of exchange agent models on a connected graph that simulates a social network, finding different distributions in each case [34].

Interactions between agents can be directly included in the exchange rules. Besides saving, risk or various social protection factors can be introduced [35]. With these interactions, one could model increasingly realistic systems and try to explain phenomena such as economic inequality based on the agents’ decisions and the policies that influence them. It would be interesting to study some economic processes defined with these generalized rules within the ensemble formalism to determine how their corresponding analogous thermodynamic variables change and eventually compare them with real economic systems. For instance, relate them to indices that characterize inequality such as the Gini or Kolkata [9,10,11]. In future works, this kind of relations will be addressed.

## Data Availability

Not applicable.
